# Advances in the Application of the Non-Conventional Yeast *Pichia kudriavzevii* in Food and Biotechnology Industries

**DOI:** 10.3390/jof9020170

**Published:** 2023-01-27

**Authors:** Yunfei Chu, Mengmeng Li, Jiahui Jin, Xiameng Dong, Ke Xu, Libo Jin, Yanming Qiao, Hao Ji

**Affiliations:** 1 Institute of Life Sciences, College of Life and Environmental Science, Wenzhou University, Wenzhou 325035, China; 2 Biomedical Collaborative Innovation Center of Zhejiang Province & Engineering Laboratory of Zhejiang Province for Pharmaceutical Development of Growth Factors, Wenzhou University, Wenzhou 325035, China; 3 Department of Agriculture and Biotechnology, Wenzhou Vocational College of Science and Technology, Wenzhou 325006, China; 4 School of Biological Science and Engineering, Shaanxi University of Technology, Hanzhong 723000, China

**Keywords:** *Pichia kudriavzevii*, food fermentation, biotechnological applications, feed additive, biological control, environmental management

## Abstract

*Pichia kudriavzevii* is an emerging non-conventional yeast which has attracted increased attention for its application in food and biotechnology areas. It is widespread in various habitats and often occurs in the spontaneous fermentation process of traditional fermented foods and beverages. The contributions of *P. kudriavzevii* in degrading organic acid, releasing various hydrolase and flavor compounds, and displaying probiotic properties make it a promising starter culture in the food and feed industry. Moreover, its inherent characteristics, including high tolerance to extreme pH, high temperature, hyperosmotic stress and fermentation inhibitors, allow it the potential to address technical challenges in industrial applications. With the development of advanced genetic engineering tools and system biology techniques, *P. kudriavzevii* is becoming one of the most promising non-conventional yeasts. This paper systematically reviews the recent progress in the application of *P. kudriavzevii* to food fermentation, the feed industry, chemical biosynthesis, biocontrol and environmental engineering. In addition, safety issues and current challenges to its use are discussed.

## 1. Introduction

Yeasts have played an indispensable role in the history of human development, with diverse applications and tremendous influence in the fields of food, biotechnology, daily chemicals and pharmaceuticals. They usually dominate in spontaneous fermented foods, contributing to desirable flavors, and have been domesticated to drive today’s industrial food fermentation as well as genetically engineered for industrial chemicals and pharmaceuticals production. Among them, *Saccharomyces cerevisiae* is regarded as a dominant workforce for bioprocesses due to its “generally regarded as safe” status, well-characterized physiology and ease of engineering [1]. However, biotechnological processing with *S. cerevisiae* faces increasing challenges, partly due to its inherent characteristics such as poor tolerance to environmental stresses and limitation in available carbon sources [2]. Moreover, the use of only *S. cerevisiae* for controlled fermentation processes limits the sensorial characteristics of products, making it difficult to meet the diversified demands of consumers [3]. These drawbacks have led to the emergence of non-conventional yeasts as attractive biotechnological hosts due to their advantageous phenotypes such as native stress tolerance, capacity to metabolize diverse carbon sources and the release of unique flavors.

**Figure 1:**
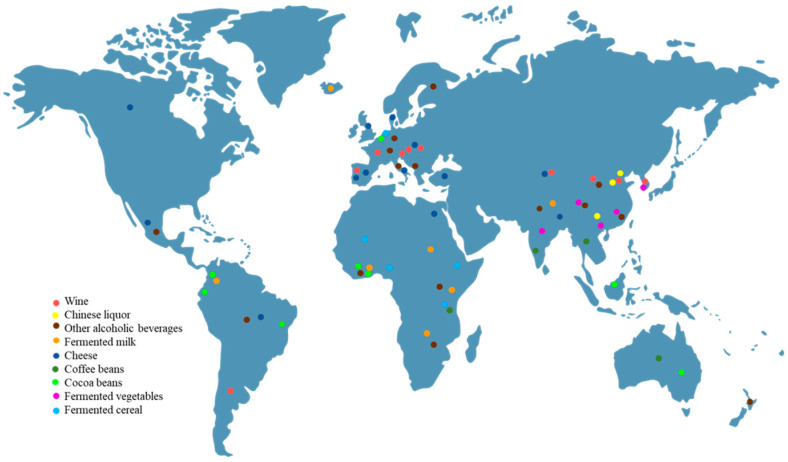
Distribution of spontaneously fermented foods involving *P. kudriavzevii* on a global scale.

*Pichia kudriavzevii*, a non-conventional yeast, is ubiquitous in natural environments and occurs in traditionally fermented foods and beverages in every continent across the globe (Figure 1). Although its safety has always been a controversial issue, it has attracted increased attention over the past two decades due to its potential application in food processing and biotechnology. It is sometimes thought to be a spoilage yeast that causes biofilm formation and odor release, but more often it contributes to the formation of flavor compounds and exhibits probiotic properties, and it has been now used as a starter culture, either alone or with other yeasts, to obtain fermented products with consistent quality. On account of its inherent characteristics such as high tolerance to pH, ethanol, high temperature, hyperosmotic stress and lignocellulosic inhibitors, *P. kudriavzevii* has prompted interest for industrial applications [4]. In fact, at least two platform chemicals, including glycerol and succinic acid, have successfully been commercially produced using *P. kudriavzevii* as the biotechnological host [5,6]. However, its biotechnological applications are still limited, mainly due to the knowledge gap of its genetic background and difficulties in genetic manipulation. More recently, numerous studies have focused on the mining of multi-omics information, the development of efficient genetic tools including inducible promoters, expression plasmids and gene editing systems, and the introduction of heterologous metabolic pathways, which allow *P. kudriavzevii* to be utilized as a promising biotechnological chassis for the production of high-value-added chemicals. In addition, *P. kudriavzevii* has also showed promising application prospects in environmental management and the postharvest biocontrol of fruits. However, there are few systematic reviews on its biotechnological applications, and the most recent one was published a decade ago [7]. In this review, we attempt to comprehensively summarize the current status of *P. kudriavzevii* in the food, feed and biotechnology industries. We also highlight concerns over its safety and provide some discussion of current challenges.

## 2. Taxonomy and Physiology of *P. kudriavzevii*

The brief history and taxonomy of *P. kudriavzevii* have been described in the earlier reviews [7,8]. This species, characterized by the formation of spherical ascospores, was initially named *Issatchenkia orientalis*, but was then transferred to the *Pichia* genus under the name of *Pichia kudriavzevii* by Kurtzman et al. [9]. Although the name *I. orientalis* has since been abandoned, it is still used by some researchers today, probably as a result of multiple sequence alignments during the strain identification as many rRNA gene sequences of strains using the name *I. orientalis* were uploaded to NCBI database in the early days. *Candida krusei*, often used to name clinical isolates, was initially proposed as the asexual form (anamorph) of *P. kudriavzevii*, sharing identical sequences of the D1/D2 regions of the 26S ribosomal DNA [9]. Douglass et al. employed genomic diversity analysis on strains isolated from different environments with different species names. Whole genome alignment performed on the two type strains of *C. krusei* (CBS573T) and *P. kudriavzevii* (CBS5147T) showed 99.6% nucleotide sequence identity, with collinear genomes [10]. The industrial glycerol-producing strain *Candida glycerinogenes* can also be identified as *P. kudriavzevii*, since their gene sequences, including 18S rDNA, are highly identical [10]. In the context of increasing academic studies on this species, *P. kudriavzevii* should be adopted as the only name according to the principle of “One Fungus One Name”, to avoid the use of multiple names impeding academic exchange.

*P. kudriavzevii* produces ovoid or elongated ellipsoidal cells that resemble long-grain rice, and it shows dimorphic transitions characterized by pseudohyphae growth and biofilm formation upon the induction of nutrient limitations [11,12]. We have reported that extreme low-pH stress also induces the formation of pseudohyphae and the progressive development of multicellular clusters, which is not conductive to mass transfer during fermentation [13]. This phenotype is different from that of *C. albicans*, whose hyphal morphologies are induced by neutral to alkaline pH [14]. Biofilm formation is considered to be associated with invasive growth and food spoilage and therefore has attracted attention both in the food industry and clinical research.

*P. kudriavzevii* is a crabtree-negative yeast that rapidly grows without ethanol generation under conditions with sufficient dissolved oxygen [15]. It can metabolize and utilize a variety of carbon sources with glucose as the preferred one, but there are individual differences in their utilization capacities. For example, most isolates cannot utilize xylose and sucrose as a sole carbon source, but some strains were reported to have this capability [16]. In any case, the poor xylose-assimilation has become a limiting factor for *P. kudriavzevii* being used in cellulosic ethanol production, although numerous studies have shown its high tolerance to cellulose inhibitors and other harsh conditions [2,17]. The high tolerance to environmental stresses may be one of the reasons for the occurrence of *P. kudriavzevii* in a variety of naturally fermented foods around the world and makes this species a promising robust platform for chemical biosynthesis.

## 3. Application in Fermented Foods and Feed Industry

### 3.1. Fermented Alcoholic Beverages

#### 3.1.1. Wine

*P. kudriavzevii* is a representative non-*Saccharomyces* yeast present in vineyard and winery environments which has a positive influence on the spontaneous wine fermentations, contributing to the improvement of aromatic profiles and organoleptic complexity. It was reported to be the fourth most common non-*Saccharomyces* yeast isolated from must samples in 2016, a year of port wine with recognized outstanding quality [18], suggesting a potential role of *P. kudriavzevii*. Its addition into must had no adverse effect on the growth of *S. cerevisiae* and the physicochemical properties of wines, but increased the content of esters and fatty acids, probably due to the production of various hydrolases [19]. Moreover, *P. kudriavzevii* degrades malic acid, which is detrimental to wine quality when present in high amounts in grapes [20,21]. The tolerance of *P. kudriavzevii* to high sugar concentrations is a favorable trait for the production of sweet wine using overripe Cabernet Sauvignon grapes. Its co-fermentation with *S. cerevisiae* reduced the residual sugar and acid content, and produced more intense floral and caramel odors [22]. These studies showed the great potential of *P. kudriavzevii* in the winemaking industry, mainly reflected in its sugar tolerance and deacidification characteristics, as well as its contribution to flavor diversity.

#### 3.1.2. Chinese Liquor

*P. kudriavzevii* participates and plays a major role in the fermentation process of traditional Chinese liquor, a popular distilled liquor for thousands of years. It was identified as a dominant functional yeast during the fermentation of Chinese light-flavor, strong-flavor, miscellaneous-flavor and sauce-flavor liquor [23,24,25,26]. *P. kudriavzevii* may have originated mainly from Daqu, the fermentation starter which usually contributes 61.1 to 80.0% of fungal communities during liquor fermentation [27]. Source-tracking analysis of microbial communities in Daqu confirmed that *P. kudriavzevii* mainly originated from its production environments, including indoor floors and tools [28]. Correlation analyses between microorganisms and metabolites showed that *P. kudriavzevii* primarily participated in the formation of ethanol and some flavor compound precursors such as acetate and acetaldehyde [29]. In addition, *P. kudriavzevii* exhibits the capacity to degrade lactic acid, which is usually accumulated during liquor fermentation, thereby facilitating the growth of *S. cerevisiae* and ethanol production [30]. Its contribution to liquor fermentation makes it a potential starter to improve the quality and stability of liquor products, although such controlled liquor fermentation is still far from being realized.

#### 3.1.3. Other Alcoholic Beverages

*P. kudriavzevii* is also found in the fermentation of other alcoholic beverages, including beer, fermented fruits or berries, and traditional cereal beverages. The increasing demand for new beers with specific characteristics has stimulated a shift in beer brewing from monoculture, using *Saccharomyces* species, to co-culture with non-conventional yeasts to alter the aromatic profile and therefore improve sensory quality. *P. kudriavzevii* was used as an aromatic starter in Côte d’Ivoire and co-fermented with *S. cerevisiae* to obtain beers with lower alcohol content and relatively more esters [31,32]. Alcoholic beverages fermented from diversified fruits and berries are also attracting more and more young consumers because of their novel and unique flavors. Fermentation with *P. kudriavzevii* produced higher amounts of ethyl acetate than that with *S. cerevisiae*, and endowed unique aromas such as bitter almond and cheese aroma [33,34]. In addition, its co-fermentation with other yeast species confers potential probiotic properties. For example, its co-fermentation products with *Wickerhamomyces subpelliculosus* using cornelian cherry fruits positively affected the composition of intestinal microbiota in an in vitro model of gastrointestinal tract digestion [35]. However, the role of *P. kudriavzevii* in the fermentation of traditional alcoholic beverages is sometimes negative. Biofilm, a characteristic associated with spoilage of beverages, forms on the surface of obutoka and Kombucha, two traditional alcoholic beverages [36,37]. Nonetheless, *P. kudriavzevii* is one of the non-*Saccharomyces* yeasts that has received the most attention in the fermentation process of alcoholic beverages in recent years. Studies on its interactions with other microbial communities and contribution to flavor profiles are essential for the process control and standardized production of alcoholic beverages.

### 3.2. Dairy Products 

#### 3.2.1. Fermented Milk

*P. kudriavzevii* has been isolated from several indigenous fermented milk products from different areas, although it was reported to be lactase-negative [38,39,40,41]. Several studies highlighted the spoilage potential of *P. kudriavzevii* because of its film-forming property under acidic conditions, and the release of alcohols and esters thought to be associated with off-flavors of fermented milk beverages [42,43]. On the other hand, *P. kudriavzevii* from a traditional fermented cow milk in Colombia was reported to produce peptides with Angiotensin I converting enzyme (ACE) inhibitory activity, which has the effect of lowering blood pressure [44]. Co-culture of *P. kudriavzevii*, *Lactobacillus plantarum* and *Enterococcus faecalis* was proved to be the most effective combination for making fermented milk with higher ACE inhibitory activity [45]. However, biofilm formation and off-flavor reduce people’s willingness to consume, so *P. kudriavzevii* is clearly undesirable for naturally fermented milk, and its population dynamics should be monitored for process control.

#### 3.2.2. Cheese

The microbial diversity and dynamics throughout the manufacturing and ripening process of cheeses have a comprehensive influence on the safety, quality and organoleptic characteristics of cheese products, and thus have been the focus of research. *P. kudriavzevii* was encountered in various cheese types, probably originating from the milk and cheeses-making environments. Lavoie et al. reported that *P. kudriavzevii* in raw cow milk could survive the manufacturing process and affect cheese ripening [46]. *P. kudriavzevii* plays a positive role in the development of the cheese flavor; it displays extracellular protease and lipase activities, which may contribute to the formation of texture and characteristic flavors [47,48]. It was reported to produce a range of aroma compounds and provided pleasing flavor such as brandy, herbaceous, and onion flavors when used as an auxiliary starter for Kazak cheese [47,49]. Some *P. kudriavzevii* strains isolated from cheeses exhibited potentially probiotic properties such as high hydrophobicity, antimicrobial activity, autoaggregation and the ability to survive the gastrointestinal tract [50,51]. However, *P. kudriavzevii* has also been recognized in certain regions as a spoilage yeast for cheesesthat cause off-flavors and texture alterations [52,53]. This difference in perception may be explained in part by regional preferences for cheese texture and flavor.

### 3.3. Coffee Beans and Cocoa Beans

Wet fermentation is one of the primary coffee processing steps in which de-mucilage is carried out through microbial action [54]. *P. kudriavzevii* was identified as one of the predominate yeasts throughout the wet fermentation of Australian coffee beans [55,56]. Unlike the traditional perception, *P. kudriavzevii* had no significant effect on the mucilage degradation but contributed to the generation of several aromatic compounds [57,58]. Inoculation fermentation of *P. kudriavzevii* increased the levels of ketones, pyrazines, and furans of roasted beans which endowed coffee with desirable sensory characteristics [59]. Recently, *P. kudriavzevii* was also isolated from the fermentation process of Arabica coffee, which has an enhancement effect on sensory impact and quality [60,61].

Similar to what occurs in coffee bean processing, microbial fermentation is also an essential step in cocoa bean processing, which strongly influences the formation of precursors for chocolate flavor and aroma [62]. *P. kudriavzevii* dominates this spontaneous fermentation process in some cocoa-producing regions and can survive even after drying [63,64]. It was well-adapted to the high temperature conditions during cocoa fermentation in the Agneby-Tiassa region, and exhibited high pectinolytic activities in acidic conditions at the early stage of fermentation, thus degrading the pulp of the cocoa bean [65]. Compared with spontaneous fermentation, mono-fermentation of *P. kudriavzevii* or co-fermentation with *S. cerevisiae* can accelerate the fermentation process and modulate the chemical characteristics of cocoa beans [66,67]. In addition, *P. kudriavzevii* has a significant degradation effect on toxic compounds such as biogenic amines, reflecting the significance of its application in food safety [68]. These laboratory scale studies demonstrated that the introduction of *P. kudriavzevii* as a starter culture is a promising method to enhance fermentation processes and achieve controlled fermentation of cocoa beans [66,67,68]. However, before that can be fully implemented, some parameters that affect the phenotypes of *P. kudriavzevii* during processing and challenges in technical scale-up need to be addressed.

### 3.4. Fermented Vegetables

*P. kudriavzevii* is considered to be a major spoilage yeast responsible for the developing of off-odor and texture softening of kimchi, a traditional Korean fermented vegetable [69]. It exhibits high polygalacturonase activity which catalyzes the hydrolysis of pectin, and thus decreases the hardness of over-ripened kimchi. Inoculation with *P. kudriavzevii* in kimchi preparation produced odor compounds, including methanethiol, that further confirmed its identity as a spoilage yeast [69]. Moreover, the formation of white colonies on the surface of kimchi has caused consumer complaints and concerns about safety issues, although a recent study confirmed the safety of *P. kudriavzevii* ingestion via kimchi consumption [70]. Nonetheless, to avoid economic losses, controlling *P. kudriavzevii* growth in kimchi is an important subject to extend the shelf life and reassure customers.

### 3.5. Fermented Cereals

Traditional cereal-based fermented foods are particularly important in providing adequate calories and nutrients in some African countries where resources are relatively scarce. *P. kudriavzevii* usually exhibits probiotic properties as well as high folate-producing capacity and is used as a starter culture for fermented cereals to overcome the heterogeneity of the products and improve the nutritional value [71,72]. Greppi et al. reported that the co-culture of *P. kudriavzevii* and *Lactobacillus fermentum* in pearl millet porridge produced high quantities of folate, showing the nutrient enrichment effect [73]. Ogunremi et al. reported the role of *P. kudriavzevii* OG32 in the increasing of flavor compounds and antioxidant activity of fermented cereals [74], and highlighted its ability to secrete phytases, demonstrating its potential to improve the bioavailability of minerals in fermented cereal [75]. Therefore, the probiotic and nutritional enrichment properties of *P. kudriavzevii* make it a promising candidate as a functional starter culture for cereal-based fermentation.

### 3.6. Applications as a Feeding Additive

The application of rumen-native microorganisms as a feed additive is an effective strategy to improve feed efficiency and animal performance. *P. kudriavzevii* was reported as a dominant yeast in bovine rumen that can survive in simulated ruminal fluid and produce cellulase and large amounts of biomass, making it a better microbial additive than the commonly used *S. cerevisiae* [76,77]. It is reported that application of *P. kudriavzevii* improved the quality of ensiled rice straw, although an earlier study on corn silage showed that high populations of *P. kudriavzevii* had an adverse effect on fiber digestibility [78,79]. A feeding study showed that supplementation of *P. kudriavzevii* T7 powder could improve milk yield and reduce somatic cell counts, hence improving dairy production efficiency [80].

Additionally, *P. kudriavzevii* as a feed additive has the advantage of degrading mycotoxins, which are present in most feedstuffs and cause severe health problems after being consumed by animals, and which can even be transferred and excreted into raw milk, leading to food safety issues. Feeding experiments revealed that dietary supplementation of *P. kudriavzevii* YSY2 reduced the conversion of aflatoxin B1 from feed into aflatoxin M1 in raw milk, and mitigated the negative effects of aflatoxin B1 on dry matter intake and milk composition [81]. *P. kudriavzevii* strains isolated from broilers’ feedstuff also showed a high adsorption capacity for aflatoxin B1, which was then used as a feed additive to alleviate the toxic effect on broilers [82,83]. The commercial potential of *P. kudriavzevii* as a feed additive needs to be explored by more basic research, such as the determination of feeding dosage and incorporation with other yeasts. Recently, Native Microbials Inc. had submitted a request to the Food and Drug Administration’s (FDA, Silver Spring, MD, USA) Center for Veterinary Medicine (CVM) to evaluate the use of *P. kudriavzevii* as a direct-fed microbial in diets of dairy cattle, but this assessment has been since ceased due to safety concerns (FDA, Silver Spring, MD, USA, 2022).

## 4. Applications in Biotechnology

### 4.1. Biochemicals Production

The application of the model host *S. cerevisiae* for next-generation chemical production faces increasing technical challenges which are difficult to overcome, in part due to its inherent limitations. Non-conventional yeasts possess potential advantages over *S. cerevisiae* such as broad substrate spectrum, inherent physiology characteristics and ability to tolerate harsh growth conditions, and their bioprocess applications have made remarkable progress with the development of systems biology and synthetic biology. Table 1 provides an overview of *P. kudriavzevii* and other well-studied non-conventional yeasts and their industrially relevant phenotypes. The desirable intrinsic phenotypes of *P. kudriavzevii* have attracted extensive attention in the production of second-generation bioethanol, glycerol and organic acids, etc. Here we summarize the current research status of this promising biotechnological host in chemical bioprocessing.

#### 4.1.1. Bioethanol

The most widespread usage of *P. kudriavzevii* in biotechnology is in the production of bioethanol due to its inherent characteristics of high ethanol tolerance and desirable ethanol production capacity. *P. kudriavzevii* outperforms some industrial *S. cerevisiae* strains in terms of fermentation efficiency under harsh conditions such as high temperature, low pH and the presence of lignocellulosic inhibitory compounds, thus exhibiting great potential for second-generation bioethanol production. Numerous studies have reported its applications in bioethanol production from renewable substrates such as rice straw, sugarcane bagasse, corncob, food waste, etc. Table 2 summarizes the performance of *P. kudriavzevii* in bioethanol production under diverse processes.

Ethanol fermentation at high temperatures offers substantial benefits such as lower cooling costs, reduced risk of contamination and cost of saccharification enzymes during simultaneous saccharification fermentation (SSF) [2,91]. *S. cerevisiae* used in the traditional ethanol industry has high production rates and tolerance to ethanol, but comparatively low fermentation temperature (25–35 °C). By contrast, *P. kudriavzevii* shows better cell growth and ethanol production capacity above 40 °C, and some isolates can ferment at up to 45 °C; thus, it is considered to be another promising thermotolerant yeast species in addition to *Kluyveromyces marxianus*. For instance, *P. kudriavzevii* HOP-1 produced 10%, 35%, and 200% more ethanol than an industrial *S. cerevisiae* strain at 35, 40 and 45 °C, respectively [92]. *P. kudriavzevii* MBY1358 produced 107.33 g/L ethanol from 200 g/L glucose within 40 h at 44 °C [93]. Metabonomics analysis revealed that the overproduction of unsaturated fatty acids, pyrimidines and purines might contribute to its thermotolerance trait [94]. Exogenous addition of NaCl was proven to be an effective approach for improving the biomass and ethanol yield of *P. kudriavzevii* at 45 °C, and this inorganic salt cross-protection effect may be attributed to the induction of key enzymes and intermediates in carbohydrate metabolism and the reduction in oxidative damage [95]. These studies provide important engineering targets for further improvement of bioethanol production at high temperatures by genetically modified *P. kudriavzevii*.

The acid-tolerance trait of *P. kudriavzevii* allows ethanol fermentation under low-pH conditions without the application of neutralizing agents, resulting in lower production costs. Seong et al. reported that the ethanol yield of *I. orientalis* MTY1 at pH 3.0 was similar to that of *S. cerevisiae* D452-2 at its optimum pH condition of 6.0 [94]. The combined effects of low-pH and salt stresses on the ethanol production were also evaluated. *I. orientalis* MF-121 produced 5.3% *v*/*v* ethanol from the medium containing 50 g/L Na_2_SO_4_ at pH 3.0, while *S. cerevisiae* K-7 could not grow under the same condition [96]. Overall, these instances suggest that *P. kudriavzevii* is a more promising biotechnological host than *S. cerevisiae* for low-pH ethanol production, mainly benefiting from its low-pH tolerant trait.

A large amount of research has focused on the bioethanol production of *P. kudriavzevii* from widely available and inexpensive renewable agricultural resources. Several *P. kudriavzevii* strains with sucrose assimilation ability were employed to ferment sugarcane juices, which is rich in sugars, organic nutrients and minerals, and is an ideal substrate for ethanol fermentation [97,98]. Galactose adaptation of *P. kudriavzevii* can produce 30% more ethanol than non-adapted cells from sugarcane juices, probably due to enhanced sugar uptake [98]. *P. kudriavzevii* has multiple tolerance to lignocellulosic inhibitors such as furfural, acetic acid and hydroxymethylfurfural, and can even ferment non-detoxified hydrolysates to produce ethanol. For example, the ethanol yield of *I. orientalis* KJ27-7-fermented wheat straw hydrolysate reached 97% of the theoretical value [99]. Kwon et al. reported that there was no significant difference in ethanol productivity using *I. orientalis* IPE100 between fermenting detoxification and non-detoxification steam-exploded cornstalk [100]. The robust physiology of *P. kudriavzevii* also confers its potential application in simultaneous saccharification and fermentation (SSF). In the SSF of alkali-treated rice straw, *P. kudriavzevii* HOP-1 produced 35% and 200% more ethanol than *S. cerevisiae* at 40 and 45 °C, respectively [92].

**Table 2:** Ethanol production by *P. kudriavzevii* strains under various conditions.

Strains	Substrates	Ethanol Production	Fermentation Conditions	References
MF-121	Glucose	5.50% (*v*/*v*)	10 g/L Na_2_SO_4_, pH 2.0	[96]
RZ8-1	Glucose	69.9 g/L, 1.46 g/L·h	40 °C	[101]
RZ8-1	Sugarcane bagasse hydrolysate	33.8 g/L, 1.41 g/L·h	40 °C	[101]
IPE 100	Cornstalk hydrolysate	45.9 g/L, 0.96 g/L·h	42 °C	[100]
IPE 100	Sweet sorghum stalk	0.25 g/g-dry stalk	Solid state fermentation	[102]
Unnamed	Sugarcane juice	71.9 g/L, 4.0 g/L·h	40 °C	[98]
ITV-S42	Glucose	66.2 g/L, 2.67 g/L·h	200 g/L initial glucose concentration	[103]
HOP-1	Alkali-treated rice straw	24.3 g/L, 1.10 g/L·h	SSF, 40 °C, pH 5.0	[92]
HOP-1	Sweet sorghum bagasse hydrolysate	26.8 g/L, 0.56 g/L·h	SSF, 35 °C, pH 5.0	[104]
KJ27-7	Wheat straw hydrolysate	10.3 g/L, 0.43 g/L·h, 0.5 g/g glucose	26 °C, pH 5.0	[99]
4A	Malted barley	115.1 g/L, 4.80 g/L·h	40 °C, pH 5.0	[105]
LC375240	Cassava peel	5.4%(*w*/*v*), 1.45 g/L·h, 0.38 g/g glucose	SSF, 40 °C	[106]
SI	Rice straw	33.4 g/L, 1.07 g/L·h	SSF, 42 °C, uncontrolled pH	[84]
KVMP10	Citrus peel waste hydrolysate	30.7 g/L	42 °C	[107]
NBRC1664	Japanese cedar without pretreatment	23.8 g/L, 0.74 g/L·h	SSF, 35 °C	[108]
DMKU3-ET15	Cassava starch hydrolysate	7.35% (*w*/*v*), 2.23 g/L·h	40 °C, pH 5.0	[109]
Pa27	Xylose	14.6 g/L, 0.27 g/g xylose	42 °C	[110]
Brk	Undetoxified acid- hydrolyzed corncob	3.64 g/L, 28% of the theoretical yield	42 °C	[111]
Brk	Detoxified acid- hydrolyzed corncob	3.93 g/L, 32% of the theoretical yield	42 °C	[111]

Note: SSF (simultaneous saccharification and fermentation); RBF (repeated batch fermentation).

#### 4.1.2. Organic Acids

Owing to its high tolerance towards low-pH stress and organic acids, *P. kudriavzevii* is considered to be an attractive host for low-pH organic acids production. A lot of efforts have been made to exploit the low pH fermentation of various organic acids using recombinant *P. kudriavzevii*, some of which have reached a commercial scale (Table 3).

Succinic acid production using *P. kudriavzevii* is already at the stage of commercialization, implemented by BioAmber and its associated firms. Several academic papers and patents have disclosed the engineering strategies for succinic acid production in *P. kudriavzevii*. There are three primary pathways of succinic acid formation in mitochondria: reductive TCA cycle, oxidative TCA cycle and glyoxylate shunt pathway, among which the reductive TCA cycle has the highest theoretical succinic acid yield [6]. In addition to expressing reductive TCA cycle-related genes in *P. kudriavzevii*, inactivation of the mitochondrial succinate dehydrogenase complex (SDH) can also be used as a simple approach to achieve succinic acid accumulation. Furthermore, deleting the carboxylic acid transporter gene *PkJEN2* prevents the re-entry of extracellular succinic acid into cells for catabolism [112]. On a commercial scale, BioAmber has several proprietary technologies for succinic acid production using recombinant *P. kudriavzevii* licensed by Cargill, and has been operating a plant at a scale of 30,000 tons per year since 2014. Their recombinant *P. kudriavzevii* can produce 48.2 g/L succinic acid at pH 3.0, with a yield of 0.69 mol/mol glucose and a productivity of 0.97 g/L/h [113]. By the application of direct crystallization might be at low pH, the unit operations in the process downstream are simplified, and with decreased investment costs. Unfortunately, BioAmber filed for bankruptcy in 2018, possibly due to the poor financial situation. However, the project for succinic acid production using *P. kudriavzevii* is still attracting investors. LCY Biosciences which purchased the assets of BioAmber claimed that their succinic acid capacity would be restored to 30,000 ton per year by 2023. Cargill also holds a patent for lactic acid production using engineered *P. kudriavzevii*, however, the productivity cannot meet the commercial demand [114]. A recent study reported a robust *P. kudriavzevii* strain that produced 135 g/L and 154 g/L of D-lactic acid at pH 3.6 and pH 4.7, with yields of 3.66 g/L/h and 4.16 g/L/h, respectively, after genetic engineering and adaptive evolution to improve lactic acid tolerance [85]. Compared with *S. cerevisiae*, which has been used for low-pH lactic acid production [115], *P. kudriavzevii* exhibited greater potential for commercial application; nonetheless, there is still a big margin for further improving the economic feasibility of lactic acid production.

*P. kudriavzevii* can be designed to oxidize xylose to xylonate by simple genetic modification. By expressing the heterogenous D-xylose dehydrogenase coding gene, the recombinant strain produced up to 171 g/L of D-xylonate at pH 5.5, and 146 g/L of D-xylonate at pH 3.0 [116]. The efficient production of D-xylonate under low-pH is attractive for industrial-scale production, and the economic viability could be further improved by using lignocellulosic biomass as feedstock. In our previous study, to improve the comprehensive utilization of sugars present in lignocellulose, we integrated xylose and glucose fermentation in an engineered *P. kudriavzevii* by a two-stage strategy whereby xylonate and ethanol were sequential produced from non-detoxified acid-pretrated corncob at low pH [117]; this provides a promising approach for reducing the overall cost of lignocellulose biorefinery. *P. kudriavzevii* is has also been genetically engineered to produce itaconic acid, which is currently produced by the filamentous fungus *Aspergillus terreus*. An engineered strain YB4010 was constructed by the CRISPR-Cas9 system, which produced 1232 mg/L itaconic acid in fed- batch fermentation without pH control [118]. The yield of itaconic acid in this study was still at very low levels, but the researchers provided effective genetic manipulation tools and proposed potential avenues for future endeavors to increase itaconic acid production.

**Table 3:** Summary of organic acids production by engineered *P. kudriavzevii*.

Organic Acids	Strains	Metabolic Engineering Strategies	Production	Fermentation Conditions	References
Succinic acid	SD108	Overexpression of endogenous genes *PYC*, *MDH*, *FUMR* and an exogenous gene *FDR* from *S.cerevisiae*	11.6 g/L, 0.12 g/g, 0.11 g/L·h	30 °C, uncontrolled pH	[119]
	CY902	Deletion of *SDH5*, deletion of *PkJEN2-1* and *PkJEN2-2*	3.60 g/L	30 °C, uncontrolled pH	[112]
	13171	Deletion of *CYB2a*, heterologous introduction of *FRD1* from *S. cerevisiae*, *MDH* from *Z. rouxii*,*FRD1* from *T. brucei*, and overexpression endogenous genes of *FUM1* and *PYC1*	23.0 g/L, 0.26 g/L·h	30 °C, pH 3.0	[120]
	13723	Deletion of *URA* and *PDC*, heterologous introduction of *MAE* from *S. pombe*, *FRD* from *Leishmania mexicana*,*MDH* from *Rhizopus delemar*, and overexpression endogenous genes of *FUM1* and *PYC1*	48.20 g/L, 0.45 g/g glucose, 0.97 g/L·h	30 °C, pH 3.0	[113]
Lactic acid	CD1690	Deletion of *CYB2* and *GPD1*, and heterologous introduction of *LDH* from *Lactobacillus helveticus*	88 g/L, 2.6 g/L·h	38–40 °C, pH 5.5	[114]
	NG7	Replacing PDC1with LDH derived from Lactobacillus plantarum	① 135 g/L, 3.66 g/L·h ② 154 g/L, 4.16 g/L·h	① 30 °C, pH 3.6 ② 30 °C, pH 4.7	[85]
Itaconic acid	YB4010	Deletion of *ICD*, heterologous introduction of *CAD* from *A. terreus*, and overexpression of Pk-*mttA*	1232 mg/L, 29.0 mg/g-glucose, 51 g/L·h	30 °C, uncontrolled pH	[118]
D-xylonate	VTT C-79090T	Heterologous introduction of *xylB* from *Caulobacter crescentus*	① 146 g/L, 1.2 g/L·h ② 171 g/L, 1.4 g/L·h	① 30 °C, pH 3.0 ② 30 °C, pH 5.5	[116]

#### 4.1.3. Glycerol

It is believed that osmotolerant yeasts accumulate large amounts of glycerol as an osmotic protectant to allow their growth under hyperosmotic conditions [5]. *P. kudriavzevii* is an osmotolerant yeast that can grow well in media containing more than 48% (*w*/*v*) glucose or 1.5 M NaCl [4], and thus has great potential for glycerol production. Indeed, as early as the 1970s, *P. kudriavzevii* was employed for glycerol production at a commercial scale in China, under the name of *C. glycerinogenes* [121]. By fruitful studies in microbial breeding and systematic optimization of the fermentation process, the yield of glycerol on an industrial scale was improved to more than 120 g/L with conversion efficiency of up to 58% using acid-hydrolyzed corn starch as substrate [121]. Moreover, *P. kudriavzevii* is also used for glycerol production under the name of *C. krusei* [122,123,124]. In the last decade, the enthusiasm for glycerol production by osmotolerant yeasts has gradually decreased, since large amounts of crude glycerol were co-produced in the biodiesel industry, marking it less economical. Nonetheless, the production of glycerol using *P. kudriavzevii* still deserves attention. *P. kudriavzevii* produces fewer by-products during glycerol fermentation, making the refining process easier and more economical [121], which can meet the growing demand for high-purity glycerol in pharmaceutical markets.

#### 4.1.4. 2-Phenylethanol

The high cytotoxicity of 2-phenylethanol to cells is a bottleneck for its microbial production, while *P. kudriavzevii* exhibits higher 2-phenylethanol tolerance than other yeast species. Fan et al. reported a *P. kudriavzevii* strain that produced 5.09 g/L 2-phenylethanol in shake flasks, which was higher than most reported strains [125]. Oscar et al. explored the potential for more economical and sustainable production of 2-phenylethanol from *P. kudriavzevii* by solid-state fermentation using various agro-industrial wastes [126,127]. Although the current titers of 2-phenylethanol are far from sufficient for industrial production, *P. kudriavzevii* is still a promising platform for the production of 2-phenylethanol with high efficiency and at low cost. Applying diversified rational or irrational engineering to improve the 2-phenylethanol tolerance and metabolic engineering to enhance the flux of 2-phenylethanol synthesis are the main directions of future efforts.

#### 4.1.5. Single-Cell Oils

Single-cell oils of oleaginous microorganisms have great potential as raw materials for biodiesel production due to their high productivity and economic feasibility. The oleaginous character of *P. kudriavzevii* was first described by Sankh et al. [128]. In a fed-batch fermentation using crude glycerol as a carbon source, the oil yield reached 23% of the cell dry weight. By contrast, while the oil-producing capacity of *P. kudriavzevii* is obviously not desirable, oleaginous yeasts such as *Rhodosporidium toruloides* and *Yarrowia lipolytica* can accumulate lipids in excess of 50% of the cell dry weight [129]. Encouragingly, another study on *P. kudriavzevii* reported a striking lipid content of 75.7% when glucose was used as the carbon source at a C/N ratio of 120/2, which is much higher than that in the first report [128,130]. It is noticeable that the use of high-value carbon sources like pure glucose is not economically feasible. Bettencourt et al. reported that *P. kudriavzevii* can utilize a wide range of cheap, waste-derived substrates rich in volatile fatty acids for oil production, such as the effluent filtrate from the anaerobic digestion of organic wastes [131]. Compared with model oleaginous yeasts, studies on lipids production of *P. kudriavzevii* are rare and relatively preliminary, in particular, none have explored the improvement of lipid content and composition by genetic engineering. With the development of genetic manipulation technology, *P. kudriavzevii* deserves more attention as a promising microbial oil factory in the future.

### 4.2. Applications in Environmental Management

Yeast cells are promising biosorbents that can be used for heavy metal removal from wastewater. *P. kudriavzevii* exhibited better performance in the bioaccumulation of cadmium (Cd), chromium (Cr), lead (Pb) and zinc (Zn) than *S. cerevisiae*, especially under complicated conditions such as low pH and high salinity [132,133]. The bioaccumulation capacity for heavy metals might highly depend on the robustness of the yeast cells, and some effective measures have been developed for improving their tolerance to heavy metals. For example, acid treatment was reported to improve the Cd resistance through cross-protection, resulting in an enhanced Cd removal rate [134]. Pollution sources of the mining areas and electroplating factories usually contain a variety of heavy metals, but few studies have focused on the combined impact of various metals on *P. kudriavzevii*. It was reported that *P. kudriavzevii* maintained its membrane integrity after treatment with Cd, Pb and Zn mixtures for 3 h, but began to lose it after 6 h due to the accumulation of intracellular reactive oxygen species [135]. To facilitate their separation from effluent after bioremediation, an immobilization method on *P. kudriavzevii* was also developed. However, all these studies are still confined to the laboratory-scale, and further studies are required in various aspects, such as the improvement of bioaccumulation capacity, construction of biosorption modeling and development of novel immobilization techniques, for real-world applications to be considered.

*P. kudriavzevii* also has potential applications in the decolorization of molasses and textile wastewater. The strain No. SF9-246 was used to decolorize the melanoidin pigment in molasses wastewater by bioadsorption and biodegradation, and is considered more suitable for practical applications due to its growth and decolorization capability under acidic conditions [136]. It was also reported to decolorize textile dyes such as Blue RR-BB, Red 7B-HE and C.I. Basic Blue 41 [137,138]. *P. kudriavzevii* CR-Y103 decolorizes BB41 at a rate of 100% within 12 h and, importantly, the degraded metabolite was nontoxic [138].

*P. kudriavzevii* is reported to be one of the dominant fungal species during the early stage of composting, which is a promising technology for reclaiming organic waste materials as agricultural fertilizers [139]. It has been used as an inoculum to degrade the organic acid to increase the pH of raw compost material, thereby accelerating the progression of composting by enhancing the activity of the bacterial community [140]. However, organic acids might accumulate and inhibit the organic matter degradation due to the death of yeast cells caused by the elevated temperature during the thermophilic phase of composting. On the basis of the heat-resistant properties of *P. kudriavzevii*, Nakasaki et al. proposed a strategy of maintaining the temperature at 40 °C for 2 days after inoculation during the self-heating phase of composting, which was effective in accelerating the degradation of organic matter and enhancing bacterial activities [141].

### 4.3. Biological Control

*P. kudriavzevii* has been identified as an antagonistic yeast that might act against postharvest decay and mildew of fruits caused by fungal pathogens via multiple mechanisms of actions. Bleve et al. first reported the killer phenomenon of *P. kudriavzevii* against the ochratoxin A producer *Aspergillus carbonarius* and *Aspergillus niger* [142]. It showed the highest ability to produce killer toxins among several endophytic yeasts isolated from apple fruits [143]. However, purified killer toxin showed high activity against bacteria but low or no activity against fungi including *Aspergillus* sp., *Penicillium* sp. and *Candida* sp. [144], suggesting that the preventive effect of *P. kudriavzevii* on fungal infections may be attributed to other mechanisms of action. In vitro and in vivo biocontrol assays against *P. digitatum* confirmed that multiple mechanisms, including biofilm formation, competition for nutrients, and production of volatile organic compounds (VOCs), were employed by *P. kudriavzevii* to control green mold in citrus [145].

Morphological changes in yeasts occurring under abiotic stresses affect their biocontrol performance as the mode of life may influence the biology and interactions with pathogens and the plant hosts. Chi et al. reported that the biofilm formation of *P. kudriavzevii* increased its tolerance to environmental factors, including heat and oxidative stress, resulting in a higher biocontrol efficacy against postharvest diseases on pear fruit than the yeast-like form [146]. Thus, increasing biofilm formation by the application of a quorum-sensing molecule such as phenylethanol may be a point of interest for further improving the biocontrol effectiveness of *P. kudriavzevii*. VOCs emitted by biocontrol yeasts are considered to be applicable for controlling postharvest pathogens during storage under airtight conditions. A recent study revealed that VOCs produced by *P. kudriavzevii* were effective against a broad spectrum of molds, and volatile compounds with antimicrobial activity such as phenylethyl acetate and phenylethanol were identified [147].

The application of *P. kudriavzevii* may also disrupt the community succession because of the competition of nutrients and space among biocontrol yeasts, pathogenic microorganisms and other native epiphytic microflora on fruit surfaces. Liu et al. analyzed the effects of *P. kudriavzevii* treatment on microbial composition and population dynamics of cherry tomato surfaces during cold storage [148]. In this case, *P. kudriavzevii* was the dominant species at the initial storage period, but its relative abundance gradually decreased during the whole storage process, while the succession of pathogens was disrupted and the abundance of several other antagonistic yeasts increased, indicating that the biocontrol effectiveness of *P. kudriavzevii* relies on the altering of the fungal population community, but not on its competitive advantage [148]. In contrast, in another study highlighting its role in nutrition and space competition, the energy metabolism and nutrient utilization efficiency of *P. kudriavzevii* were enhanced by the synergistic promotion effect of calcium ascorbate treatment, resulting in improved biocontrol efficacy against cherry tomato gray mold [149]. Overall, similar to other antagonistic yeasts, multiple mechanisms cooperatively work in the biological control of *P. kudriavzevii*, and the complex interactions among the host, microbiome and environment remains to be further elucidated. Indeed, the application of *P. kudriavzevii* faces many obstacles, and is still insufficiently developed to be commercially available.

## 5. Pathogenicity of *P. kudriavzevii*

The great application of *P. kudriavzevii* in the field of food and biotechnology cannot conceal the fact that it is an opportunistic pathogen. *P. kudriavzevii* has long acted as a villain under the name of *C. krusei*, responsible for 2.8% of yeast infections in humans [150]. However, the pathogenicity of *P. kudriavzevii* is rarely mentioned by the researchers involved in food and biotechnology fields, possibly due to the use of different species names and less academic exchange between them and medical researchers. Strains isolated from clinical samples by medical researchers are often designated *C. krusei*, while the environment isolates used in food and biotechnology fields are referred to as *P. kudriavzevii* or *I. orientalis*. *P. kudriavzevii* sometimes inhabits the intestines of healthy individuals, but causes significant morbidity and mortality in immunocompromised patients, including premature neonates, cancer patients, and hematologic malignancy patients [151,152,153,154]. Cases have shown that *P. kudriavzevii* is responsible for peritonitis, endophthalmitis, asthma, osteomyelitis and other diseases [8]. Recently, *P. kudriavzevii* has also been isolated from hospitalized COVID-19 patients with oropharyngeal candidiasis, which requires attention in the context of the COVID-19 pandemic [155].

Compared with other *Candida* species, fewer yeast infections are attributed to *P. kudriavzevii*, but its inherent resistance to fluconazole, the most commonly used antifungal agent for the treatment of yeast infection, has attracted increasing attention in recent years. Crucially, strains isolated from food environments exhibited high resistance to fluconazole, as did strains isolated from clinical samples, suggesting that *P. kudriavzevii* used in food and biotechnology industries could also infect immunocompromised populations [10]. Although a healthy population with immunocompetence is not generally infected with *P. kudriavzevii*, the transient commensalism acquired from food or working environment might also bring potential risks. A study in Amerindians living in a remote community showed that over 30 % of the population carried *P. kudriavzevii*, probably acquired from food or living environments [156]. To escape the threat of drug resistance, effective explorations on improving the activity of existing drugs, searching for novel antifungal drugs and developing alternative treatment approaches were carried out for treating *P. kudriavzevii* infections. For example, Bianchin et al. developed fluconazole-loaded lipid core nanoparticles, and this delivery system can reverse fluconazole resistance [157]. Patriota et al. purified a trypsin inhibitor from Tecoma stans leaves, which showed anti-*Candida* activity and no cytotoxicity against human peripheral blood mononuclear cells [158]. However, the drug-resistance mechanism of *P. kudriavzevii* has not been well-understood, hindering the discovery of novel therapeutic targets and the development of new drugs, which requires further exploration.

## 6. Concluding Remarks and Future Perspective

In this review, we comprehensively summarized the current application of *P. kudriavzevii* in the food and biotechnology fields. With regards to food fermentation, *P. kudriavzevii* is often present in and dominates the spontaneously fermented foods, contributing characteristic flavors and showing probiotic potential, and can be used as a starter culture to improve the fermentation efficiency and achieve controllable food processing. The occurrence of *P. kudriavzevii* is beneficial for most fermented foods and beverages, but it is sometimes referred as a spoilage yeast due to biofilm formation and release of odors. Therefore, the application of *P. kudriavzevii* should also be distinguished for different fermented foods. For example, the *P. kudriavzevii* population should be monitored and controlled during the fermentation of milk and kimchi, while it can be used as a starter culture during the processing of cocoa and coffee beans. When it is used as a starter culture, the dosage may be an important factor affecting the quality of the fermented food, as the competition for nutrition and space has a strong influence on the microbial composition and population dynamics of the spontaneous fermentation and, further, on the texture, flavor and nutritive value of fermented products. Few studies have addressed this point, although Zhu et al. did report the influence of inoculation ratios on the flavor of fermented food during co-fermentation of *P. kudriavzevii* and *S. cerevisiae* [22]. Crucially, the safety of *P. kudriavzevii* has been highly controversial, both as an opportunistic pathogen that causes infection in immunocompromised people, and as a “generally recognized as safe” species retained in the updated inventory of microbial food cultures published by the International Dairy Federation in 2022 [159]. No genetic distinction and similar drug resistance between clinical isolates and environmental isolates suggest that traditional fermented foods may be a potential source of clinical infection. Even so, only few safety evaluations of *P. kudriavzevii* have been performed in the field of food fermentation, and numerous researchers have used this species as a starter culture for food application in recent years. Undoubtedly, it is unfeasible to prohibit the consumption of fermented foods whose fermentation process involves *P. kudriavzevii*, because spontaneous fermentation, while originally used as a reliable way of preserving food, has gradually developed into unique regional food cultures, especially in some economically backward regions. Therefore, developing novel antifungal drugs, avoiding the application of *P. kudriavzevii* in the standardized food industry, and altering immunocompromised people’s preference for traditional fermented foods are crucial for the effective management of infections. In contrast to other *Candida* species such as *C. albicans* and *C. tropicalis*, *P. kudriavzevii* has not received much attention in clinical practice, even though the abuse of antifungal drugs involving fluconazole has led to its increasing isolation from clinical samples. Future research on its drug-resistance mechanisms is crucial for the effective management of infections, and also to help in the development of novel drugs.

Inherent capabilities of industrial significance such as the tolerance to high temperatures, low pH and lignocellulosic inhibitors make *P. kudriavzevii* a promising microbial host. Its practical application in biotechnology lags far behind that of *S. cerevisiae* due to the lack of genetic manipulation tools for a long time; however, this dilemma has been recently ameliorated by the availability of higher-quality genomes, and efficient genetic tools, including episomal plasmid and CRISPR/Cas9 systems [160,161]. The establishment of these genetic tools can make it easy to control gene expression, balance metabolic flow and even introduce heterologous metabolic pathways and transport systems, which would also contribute to the study of mechanisms underpinning industrially relevant phenotypes. Omics techniques have also been widely used to explore the stress tolerance mechanism of *P. kudriavzevii*, and these can provide more valuable genetic information for the further construction of engineered strains with better traits. In addition, knowledge gaps regarding physiological mechanisms, such as the cell morphogenesis involved in biofilm formation and multicellular growth in clusters under certain conditions, should also be filled as these may be undesirable for food, industrial fermentation and the treatment of infections. Compared with other non-conventional yeasts, the inability or only slow utilization of xylose is a key bottleneck limiting the application of *P. kudriavzevii* to ethanol or other chemical production from lignocellulosic biomass. In this context, introducing heterogenous xylose metabolism and transporter systems in *P. kudriavzevii* is an essential step for further improving the economic viability of the lignocellulosic industry. Furthermore, some key metabolic pathways and transporters are still uncharacterized, limiting the application of cheap substrates such as crude glycerol. Overall, for a fruitful microbial cell factory, there is an urgent need to fill the knowledge gaps around the genetic and metabolism basis of key *P. kudriavzevii* attributes.

## Figures and Tables

**Table 1:** Overview industrially relevant phenotypes of *P. kudriavzevii* and other well-studied non-conventional yeasts.

Non-Conventional Yeasts	Glucose (*w*/*v*)	Ethanol (*v*/*v*)	Temperature (°C)	Acetic Acid (g/L)	Furfural (g/L)	5-HMF (g/L)	Low-pH	Reference
*Pichia kudriavzevii*	48%	13%	50	18	5	7 g/L	1.5	[4,17,84,85]
*Kluyveromyces lactis*	48%	10%	39	4	-	4 g/L	-	[4]
*Kluyveromyces marxianus*	40%	7%	52	15	3	7 g/L	2.5	[4,17,86]
*Yarrowia lipolytica*	50%	5%	30	5	2.9	5 g/L	-	[4,87,88]
*Candida glabrata*	50%	11%	41	-	-	7 g/L	2.0	[4,89]
*Zygosaccharomyces rouxii*	70%	13 %	30	-	-	5 g/L	2.2	[4,90]

## Data Availability

Not applicable.
